# Supplementary material for: Prolonged Consumption of A2 β-Casein Milk Reduces Symptoms Compared to A1 and A2 β-Casein Milk in Lactose Maldigesters: A Two-Week Adaptation Study
Source: Nutrients. 2024 Jun 20;16(12):1963. doi: 10.3390/nu16121963 (PMC11206509; doi:10.3390/nu16121963)
Supplement: Supplementary file 1 [file nutrients-16-01963-s001.zip › nutrients-3051060-supplementary.pdf]

nutrients MDPI

Supplementary Material

Table S1: Day-to-day changes in symptoms.

Symptoms for each day, starting from day 2 of the intervention, were compared to symptoms on day 1 and reported here as estimate ± standard error along with the p-values for the difference. The estimate indicates the change in log-odds of response variable with increase in a day. A negative estimate indicates that the log-odds of experiencing symptoms were lower by the estimate compared to day 1, and a positive estimate indicates that the log-odds of experiencing symptoms were higher by the estimate compared to day 1. NaN indicates that the value is not a number and that the log of odds could not be computed due to reasons such as division by zero.

**Abdominal pain**	**A1/A2 milk**		**A2 milk**	
	Estimate ± Standard error	P-value	Estimate ± Standard error	P-value
Day 1 ǀ Day 2	1.347404 ± 0.652947	0.0391*	-0.6310 ± 0.5877	0.2829
Day 1 ǀ Day 3	0.201540 ± 0.569623	0.7235	-0.467 ± 0.5921	0.4304
Day 1 ǀ Day 4	-0.905585 ± 0.598130	0.13	-0.4444 ± 0.582	0.4450
Day 1 ǀ Day 5	-0.259012 ± 0.673901	0.7007	0.493 ± 0.5756	0.3917
Day 1 ǀ Day 6	-0.808922 ± 0.638568	0.2052	-0.3409 ± 0.5761	0.5541
Day 1 ǀ Day 7	0.437568 ± 0.554039	0.4297	-0.0182 ± 0.5606	0.9741
Day 1 ǀ Day 8	-0.009131 ± 0.620200	0.9883	0.2483 ± 0.5526	0.6531
Day 1 ǀ Day 9	-0.073349 ± 0.722799	0.9192	0.3515 ± 0.5662	0.5348
Day 1 ǀ Day 10	1.733590 ± 0.734333	0.0182*	0.6842 ± 0.5533	0.2163
Day 1 ǀ Day 11	-0.429588 ± 0.641357	0.503	0.9005 ± 0.5384	0.0944
Day 1 ǀ Day 12	0.531629 ± 0.577176	0.357	0.3076 ± 0.5454	0.5727
Day 1 ǀ Day 13	-0.067088 ± 0.558243	0.9043	-0.0541 ± 0.5500	0.9217
Day 1 ǀ Day 14	0.229555 ± 0.555856	0.6796	-0.4439 ± 0.5477	0.4176
**Bloating**	**A1/A2 milk**		**A2 milk**	
	Estimate ± Standard error	P-value	Estimate ± Standard error	P-value
Day 1 ǀ Day 2	1.39328 ± 0.60154	0.020548 *	-1.0146 ± 0.5963	0.088
Day 1 ǀ Day 3	0.01529 ± 0.52041	0.976562	-0.4764 ± 0.595	0.4232
Day 1 ǀ Day 4	-1.89825 ± 0.55686	0.000652 *	0.0547 ± 0.5771	0.925
Day 1 ǀ Day 5	0.65458 ± 0.62046	0.291428	-0.0332 ± 0.5773	0.9542
Day 1 ǀ Day 6	-1.14657 ± 0.58451	0.049809 *	0.0544 ± 0.5947	0.9271
Day 1 ǀ Day 7	0.64566 ± 0.50849	0.204172	1.0412 ± 0.5798	0.0725
Day 1 ǀ Day 8	0.74402 ± 0.56640	0.188984	0.0352 ± 0.5733	0.9511
Day 1 ǀ Day 9	-0.96807 ± 0.66275	0.144103	0.228 ± 0.5857	0.6972
Day 1 ǀ Day 10	1.09383 ± 0.66627	0.100649	0.279 ± 0.5673	0.6228
Day 1 ǀ Day 11	-0.55675 ± 0.58467	0.340975	-0.5928 ± 0.5407	0.272
Day 1 ǀ Day 12	0.73138 ± 0.52126	0.160588	-0.462 ± 0.5492	0.4003
Day 1 ǀ Day 13	-0.51551 ± 0.49507	0.297747	-0.0107 ± 0.569	0.9852
Day 1 ǀ Day 14	-0.05172 ± 0.49914	0.91747	-0.2596 ± 0.5657	0.6463
**Flatulence**	**A1/A2 milk**		**A2 milk**	
	Estimate ± Standard error	P-value	Estimate ± Standard error	P-value
Day 1 ǀ Day 2	0.409068 ± 0.576161	0.4777	-1.0165 ± 0.5154	0.0486 *
Day 1 ǀ Day 3	0.270420 ± 0.485701	0.5777	-0.0003 ± 0.5106	0.9995
Day 1 ǀ Day 4	-0.854302 ± 0.523586	0.1028	-0.641 ± 0.5055	0.2048

Day 1 ǀ Day 5	0.117061 ± 0.612449	0.8484	0.3641 ± 0.4944	0.4614
Day 1 ǀ Day 6	-0.001565 ± 0.569671	0.9978	0.0457 ± 0.5005	0.9272
Day 1 ǀ Day 7	1.214919 ± 0.478211	0.0111 *	-0.6174 ± 0.4878	0.2056
Day 1 ǀ Day 8	-0.443881 ± 0.555622	0.4244	0.2654 ± 0.4754	0.5767
Day 1 ǀ Day 9	-0.518844 ± 0.668086	0.4374	0.5578 ± 0.4978	0.2626
Day 1 ǀ Day 10	0.065073 ± 0.669187	0.9225	-0.001 ± 0.4798	0.9983
Day 1 ǀ Day 11	-0.171231 ± 0.583367	0.7691	0.3753 ± 0.4592	0.4137
Day 1 ǀ Day 12	-0.357043 ± 0.508807	0.4829	-0.2026 ± 0.4784	0.6720
Day 1 ǀ Day 13	0.484277 ± 0.494243	0.3272	-0.4025 ± 0.483	0.4046
Day 1 ǀ Day 14	-0.537230 ± 0.479402	0.2624	-0.033 ± 0.4692	0.9439
**Diarrhea**	**A1/A2 milk**		**A2 milk**	
	Estimate ± Standard error	P-value	Estimate ± Standard error	P-value
Day 1 ǀ Day 2	-0.62971 ± NaN	NaN	0.6598 ± 0.689	0.338
Day 1 ǀ Day 3	0.96871 ± NaN	NaN	-1.1271 ± 0.6896	0.1021
Day 1 ǀ Day 4	-0.46531 ± NaN	NaN	-0.5487 ± 0.6705	0.4131
Day 1 ǀ Day 5	-0.68658 ± NaN	NaN	0.3856 ± 0.673	0.5667
Day 1 ǀ Day 6	0.13677 ± NaN	NaN	1.1965 ± 0.6860	0.0811
Day 1 ǀ Day 7	0.21815 ± NaN	NaN	-0.1162 ± 0.6574	0.8597
Day 1 ǀ Day 8	-0.01656 ± NaN	NaN	-0.7304 ± 0.6544	0.2644
Day 1 ǀ Day 9	1.41124 ± NaN	NaN	0.1435 ± 0.685	0.8341
Day 1 ǀ Day 10	-0.84670 ± NaN	NaN	-0.1042 ± 0.6573	0.874
Day 1 ǀ Day 11	2.01354 ± NaN	NaN	-0.2832 ± 0.6193	0.6475
Day 1 ǀ Day 12	-1.12466 ± NaN	NaN	-0.5318 ± 0.6400	0.406
Day 1 ǀ Day 13	-0.13796 ± NaN	NaN	-0.6555 ± 0.6421	0.3073
Day 1 ǀ Day 14	0.61989 ± NaN	NaN	0.0122 ± 0.6363	0.985
**Fecal urgency**	**A1/A2 milk**		**A2 milk**	
	Estimate ± Standard error	P-value	Estimate ± Standard error	P-value
Day 1 ǀ Day 2	1.23736 ± 0.63341	0.0508	-0.4541 ± 0.5974	0.4473
Day 1 ǀ Day 3	-0.08878 ± 0.54078	0.8696	-1.2706 ± 0.6181	0.0398 *
Day 1 ǀ Day 4	-0.69752 ± 0.58239	0.231	-0.7818 ± 0.5895	0.1848
Day 1 ǀ Day 5	0.83979 ± 0.66718	0.2081	0.2537 ± 0.5896	0.6669
Day 1 ǀ Day 6	-0.47198 ± 0.61268	0.4411	-0.1672 ± 0.5982	0.7799
Day 1 ǀ Day 7	0.23974 ± 0.50943	0.6379	0.0802 ± 0.5744	0.889
Day 1 ǀ Day 8	1.38607 ± 0.59521	0.0199 *	-1.0251 ± 0.5899	0.0823
Day 1 ǀ Day 9	-0.18465 ± 0.71770	0.797	0.7812 ± 0.5812	0.1789
Day 1 ǀ Day 10	1.65747 ± 0.72867	0.0229 *	0.9931 ± 0.5828	0.0884
Day 1 ǀ Day 11	-0.39446 ± 0.62352	0.527	0.1428 ± 0.5631	0.7999
Day 1 ǀ Day 12	0.14305 ± 0.55634	0.7971	-0.9146 ± 0.5559	0.0999
Day 1 ǀ Day 13	-1.18981 ± 0.52823	0.0243 *	0.5224 ± 0.5886	0.3747
Day 1 ǀ Day 14	0.32195 ± 0.51723	0.5336	-0.5689 ± 0.5947	0.3387
